# Skin as a Potential Entry Point for SARS-CoV-2 Virus

**DOI:** 10.3390/ijms27125382

**Published:** 2026-06-15

**Authors:** Dimitri Trubetskoy, Patrick Grudzien, Daria Chudakova, Anna Klopot, Bo Shi, Pankaj Bhalla, Bethany Perez White, Irina Budunova

**Affiliations:** 1 Department of Dermatology, Northwestern University, Chicago, IL 60611, USA; 2 Center for Precision Genetic Technologies for Medicine, Engelhardt Institute of Molecular Biology of the Russian Academy of Sciences, 119991 Moscow, Russia; 3 Division of Medical Biochemistry, Department of Biochemistry and Immunochemistry, Faculty of Medicine, Wroclaw Medical University, 50-368 Wroclaw, Poland; 4 Skin Biology and Diseases Resource-Based Center, Northwestern University, Chicago, IL 60611, USA; 5 Department of Dermatology, Rush Medical College, Chicago, IL 60612, USA

**Keywords:** SARS-CoV-2, COVID-19, ACE2, TMPRSS2, skin, skin models, inflammation

## Abstract

The primary route of SARS-CoV-2 entry is via respiratory epithelium. However, many COVID-19 patients developed dermatological lesions, and SARS-CoV-2 RNA has been detected in the patients’ skin. Inflammatory skin diseases, psoriasis and atopic dermatitis (AD), significantly increased the risk of COVID-19. To evaluate the potential role of skin in SARS-CoV-2 host interactions, we utilized 3D human skin organoids (HSO) generated from human epidermal keratinocytes, as well as neonatal skin explants. HSO were treated with cytokines involved in acute and chronic skin inflammation and cytokine storm in severe COVID-19 disease: TNF-α, IL-6, IL-1β, and IFN-γ, individually and in combination. HSO were also treated with Th1 (TNF-α + IL-17) and Th2 (IL-4 + IL-13) cocktails inducing pro-psoriasis and pro-AD HSO changes, respectively. All individual cytokines, and especially their combinations, elevated the expression of ACE2 and TMPRSS2 at mRNA/protein levels. The Th2 cocktail induced only TMPRSS2, the Th1 cocktail predominantly induced ACE2. Topically applied Spike-pseudotyped lentiviral Tomato reporter, which binds ACE2 similarly to SARS-CoV-2, successfully transduced control and cytokine-treated HSO as well as neonatal skin explants. Cytokine treatment, especially TNF-α + IL-6 + IL-1β + IFN-γ and the Th1 cocktail, significantly increased viral entry. Transcriptomic analysis further revealed partial overlap between gene expression signatures induced by Spike-mediated entry in inflamed HSO and those observed in lung tissue from COVID-19 patients, supporting the biological relevance of skin models. Together, these findings demonstrate that inflammation may transiently alter the permissiveness of human skin to SARS-CoV-2 entry, suggesting that the skin may represent a previously underappreciated, although likely limited, interface in viral- host interactions.

## 1. Introduction

The Coronavirus Disease 2019 (COVID-19), caused by Severe acute respiratory syndrome coronavirus 2 (SARS-CoV-2), has posed a major global health challenge and continues to represent a potential threat due to the ongoing emergence of SARS-CoV-2 variants with differing levels of immune evasion and pathogenicity. Despite extensive research efforts, many important aspects of SARS-CoV-2 biology remain incompletely understood, including potential alternative routes of transmission and the mechanisms underlying the diverse clinical manifestations of COVID-19.

SARS-CoV-2 is a single-stranded RNA virus whose infectivity mainly depends on the binding of the viral Spike (S) glycoprotein to the host cell receptor angiotensin-converting enzyme 2 (ACE2), followed by priming and cleavage by the host protease transmembrane serine protease 2 (TMPRSS2) [1,2].

ACE2, a monocarboxypeptidase involved in renin-angiotensin signaling, plays important roles in cardiovascular and renal physiology. TMPRSS2 is a membrane-associated protease with major functions in prostate biology and prostate cancer progression. TMPRSS2 promotes SARS-CoV-2 infection by cleaving ACE2, thereby enhancing viral uptake, and by cleaving the Spike protein itself, enabling membrane fusion and viral internalization [1,2]. There are also several other non-primary receptors and cofactors that may contribute to SARS-CoV-2 cell entry [3].

The respiratory epithelium is recognized as the primary entry point for SARS-CoV-2. However, cells in other tissues and organs including the gastrointestinal tract, kidney, heart, eye, and skin also express ACE2 and TMPRSS2 [4,5,6,7,8,9]. This broad expression pattern may underline the virus’s multi-organ tropism and contribute to the extra-pulmonary clinical manifestations observed in COVID-19 patients.

The role of barrier organs such as the gastrointestinal tract and eye as additional entry points for SARS-CoV-2 has been established [5,10], but the potential role of skin remains insufficiently investigated. Recent single-cell RNA sequencing and immunostaining studies demonstrated that ACE2 is expressed in both basal and differentiated keratinocytes and is co-expressed with TMPRSS2 in the stratum granulosum [8,11]. According to our bulk RNA-sequencing (RNA-seq) data, both *ACE2* and *TMPRSS2* rank within the top 10% of expressed genes in healthy human skin [12]. Moreover, the levels of *ACE2* mRNA in skin are somewhat comparable with its levels in lungs, the primary entry site of SARS-CoV-2 [13], highlighting skin as a potentially permissive tissue for SARS-CoV-2 infection.

Despite its efficient physical and immunological barrier properties, skin is directly infected by viruses such as Herpes simplex virus, Varicella zoster virus, and Molluscum contagiosum virus, whose entry and replication largely occur within the epidermis. There is also evidence suggesting that skin may be targeted by SARS-CoV-2. Indeed, 1–20% of COVID-19 patients develop transient or persistent skin manifestations, including chilblain-like acral lesions (pernio, also known as “COVID toes”), vasculitis-like lesions, urticaria-like lesions, and maculopapular eruptions, which can occur even before the onset of major systemic symptoms [14,15,16]. Further, viral RNA and capsid proteins of SARS-CoV-2 have been detected in skin biopsies and autopsy samples from COVID-19 patients [15,17,18].

Importantly, inflammatory skin diseases such as psoriasis and atopic dermatitis (AD) have been associated with an increased risk of COVID-19 [19,20,21]. Moreover, increased ACE2 and TMPRSS2 expression has been reported in lesional skin of psoriatic patients [20,22,23]. However, the pattern and dynamic of the changes of the ACE2 and TMPRSS2 expression under different inflammatory conditions in skin, and the role of healthy or inflamed human skin in SARS-CoV-2 entry and COVID-19 pathogenesis remains poorly understood.

The major goals of our study were to: (i) determine whether SARS-CoV-2 can enter skin cells; and (ii) assess how individual inflammatory cytokines and cytokine cocktails: Th1 (TNF-α + IL17), Th2 (IL4 + IL13), and a combination of IL-1β + IL-6 + IFN-γ + TNF-α resembling the inflammatory cytokine storm induced by SARS-CoV-2 infection, affect ACE2 and TMPRSS2 expression and virus entry. We employed two in vitro skin models: neonatal skin explants and human skin organoids (HSO) generated from neonatal keratinocytes seeded on a collagen matrix and maintained on liquid/air interface [24]. Mature HSO have well established multilayer epidermis closely resembling epidermis of human skin [24]. To visualize and quantify entry, we used a Spike-pseudotyped lentiviral Tomato reporter expressing the Spike protein from the original Wuhan-Hu-1 strain.

Given the reported higher risk of COVID-19 infection in African American (AA) populations [25,26,27], and different risk of inflammatory skin diseases such as AD and psoriasis in AA and White non-Hispanic (WNH) populations we also evaluated potential differences in ACE2 and TMPRSS2 induction by cytokines and Spike -pseudotyped lentiviral reporter entry rates in AA and WNH skin models.

## 2. Results

### 2.1. ACE2 and TMPRSS2 Are Highly Expressed in Human Skin and 3D Human Skin Organoids

**Figure 1:**
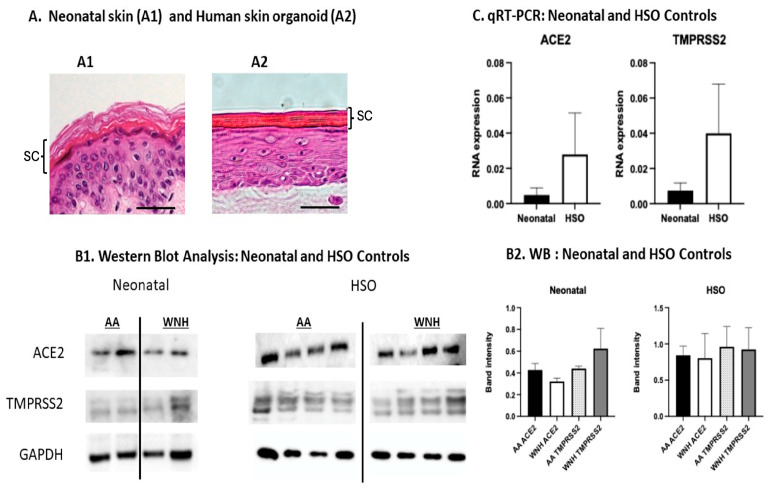
**Expression of ACE2 and TMPRSS2 in human neonatal skin and in HSO.** RNA and proteins were extracted from untreated AA and WNH neonatal foreskin and HSO epidermis. (**A**). Morphology of epidermis in neonatal skin and HSO. Hematoxylin and Eosin (H&E) staining. Scale Bar is 10 µm. SC—stratum corneum (**B1**,**B2**). ACE2 and TMPRSS2 protein expression analyzed by Western blotting in individual samples (*n* = 4 for explants and *n* = 8 for HSO). GAPDH was used as a loading control; (**C**). qRT-PCR analysis of *ACE2* and *TMPRSS2* expression. *RPL27* was used as a cDNA normalization control. Statistical analysis was done by an unpaired two-tailed Welch’s *t*-test. Error bars represent mean ± SD.

Our previously published transcriptome analysis of healthy human skin revealed robust expression of both ACE2 and TMPRSS2 at the mRNA level, with both genes ranking among the top 10% of expressed genes in human skin [12,28]. Here, we extended these findings by direct analysis of ACE2 and TMPRSS2 expression by qRT-PCR and Western blotting in neonatal skin and in 3D human skin organoids (HSO) that develop all epidermal layers including basal, spinous, dense granular layers and extensive stratum corneum critical for skin barrier function (Figure 1A).

These experiments also revealed substantial inter-individual variability in ACE2 and TMPRSS2 expression (Figure 1(B1,B2),C). We did not observe significant differences in ACE2 or TMPRSS2 expression between control AA and WNH skin explants or between control AA and WNH HSO due to a small number of samples (Figure 1(B1,B2)).

### 2.2. Proinflammatory Cytokines Strongly Induce ACE2 and TMPRSS2 Expression in HSO

**Figure 2:**
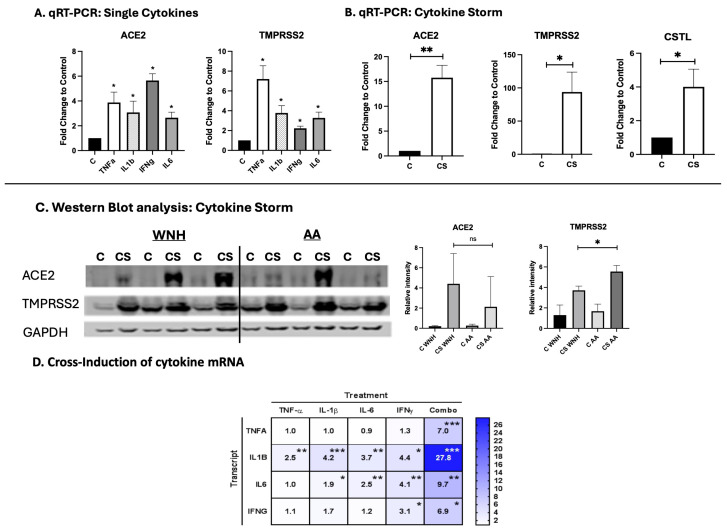
**Induction of ACE2 and TMPRSS2 in HSO by cytokines related to the COVID-19 cytokine storm.** Mature HSO made from the individual keratinocyte cell lines were treated with TNF-α, IL-6, IL-1β, and IFN-γ individually (10 ng/mL each) or in combination for 7 days. (**A**,**B**) *ACE2* and *TMPRSS2* expression was analyzed by qRT-PCR in (3–4 HSO/group). *RPL27* used as a cDNA normalization control; (**C**) Western blot analysis of ACE2 and TMPRSS2 expression. GAPDH was used as a loading control, (**D**) HSO (3–4 HSO/group made from the individual keratinocyte cell lines) were treated with the cytokine storm—related individual cytokines as in (**A**). The cross-induction of *TNF-α*, *IL-6*, *IL-1β*, and *IFN-γ* expression was assessed by qRT-PCR. The results are presented as Heatmap. Color intensity reflects fold change in mRNA expression induction compared to internal control. C—Control, CS—cytokine storm-related cytokine combination. Statistical analysis was performed by an unpaired two-tailed Welch’s *t*-test. Error bars represent mean ± SD. Statistically significant difference compared to control: *—*p* < 0.05; **—*p* < 0.02; ***—*p* < 0.01; ns—not significant.

To determine whether inflammation affects key factors central to SARS-CoV-2 entry, mature HSO cultures were treated with TNF-α, IL-6, IL-1β, and IFN-γ cytokines that play key roles in acute and chronic skin inflammation and in the cytokine storm associated with severe COVID-19 [29,30]. All four cytokines significantly upregulated ACE2 and TMPRSS2 expression at both the mRNA and protein levels, with particularly strong induction when applied in combination (Figure 2A–C). The synergistic effects of cytokine combination on ACE2 and TMPRSS2 expression reflect positive crosstalk among these cytokines, as each cytokine upregulated the expression of the others (Figure 2D).

The magnitude of induction varied significantly among individual HSO, depending on the donor source of the primary keratinocytes. The induction of ACE2 at the protein level was comparable in AA and WNH HSO, but TMPRSS2 induction was higher in AA HSO (Figure 2).

Next, we examined the effect of cytokine treatment on the expression of other reported receptors for the SARS-CoV-2 Spike protein and alternative proteases implicated in viral entry, including Kringle Containing Transmembrane Protein 1 (KREMEN1), Asialoglycoprotein Receptor 1 (ASGR1), AXL receptor tyrosine kinase, cathepsin L (CTSL), furin, and neuropilin-1 (NRP1) [31]. This analysis was performed using the RNA-seq data related to control and cytokine storm-treated HSO (**GSE304119**). We did not reveal significant changes in the expression of *AXL*, *NRP1*, and *furin*, whereas *KREMEN1* and *ASGR1* were downregulated in inflamed HSO (Supplemental Table S1). The only exception was *CTSL*, whose expression was increased approximately fivefold in HSO treated with a cytokine combination (Figure 2B).

**Figure 3:**
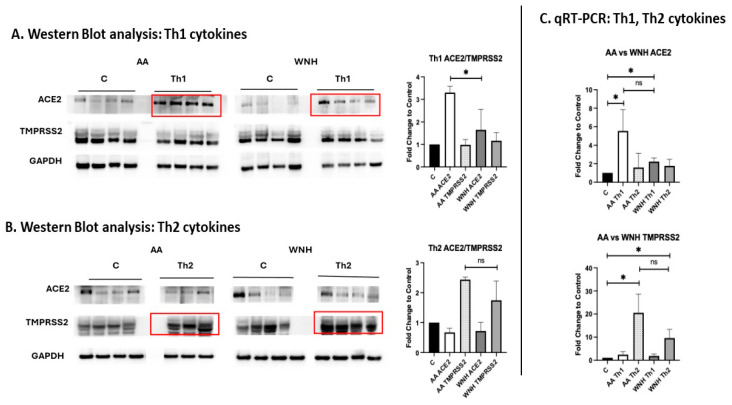
**Induction of ACE2 and TMPRSS2 in HSO by Th1 and Th2 cytokine cocktails**. Mature AA and WNH HSO (4 HSO/group) were treated with Th1 (IL-17 + TNF-α, 10 ng/mL each) or Th2 (IL-4 + IL-13, 30 ng/mL each) cytokine cocktails for 7 days. (**A**,**B**) Western blot analysis of ACE2 and TMPRSS2 expression in AA and WNH HSO after the treatment with Th1 (**A**) and Th2 (**B**) cytokines. GAPDH was used as a loading control; (**C**) qRT-PCR analysis of *ACE2* and *TMPRSS2* after Th1 and Th2 treatment, Statistical analysis was performed by an unpaired two-tailed Welch’s *t*-test. Error bars represent mean ± SD. *—Statistically significant difference compared to control (*p* < 0.05); ns—not significant. The significant differences in ACE2 and TMPRSS2 protein expression after the treatments are highlighted by red frames.

Th1 (TNF-α + IL-17) and Th2 (IL-4 + IL-13) cytokine cocktails are known to induce in HSO cultures pro-psoriasis and pro–atopic dermatitis (AD) morphological and molecular changes, respectively [24,32]. Because inflammatory skin diseases such as psoriasis and AD were associated with increased COVID-19 risk [21,33], and because disparities in the prevalence of inflammatory skin diseases have been reported between AA/Black and WNH populations [26], we assessed the effects of Th1 (pro-psoriasis) and Th2 (pro-AD) cytokine cocktails on ACE2 and TMPRSS2 protein expression in AA and WNH HSO groups (Figure 3).

The results of Western blots correlated with induction of ACE2 and TMPRSS2 expression at mRNA level. Interestingly, Th1 cytokines predominantly induced ACE2 expression, whereas Th2 cytokines primarily induced TMPRSS2 expression in HSO. Moreover, the effects of Th1 cytokine cocktail on ACE2 expression at protein level and as a trend at mRNA level were more pronounced in AA HSO (Figure 3A,B).

### 2.3. Spike-Pseudotyped Tomato/RFP Lentiviral Reporter Entry into Control and Inflamed Skin Models

The expression of ACE2 and TMPRSS2 in the neonatal skin and in HSO suggested that these skin models could be susceptible to SARS-CoV-2 transduction. To test this, we employed a biosafety level 2, non-replicating Tomato/RFP fluorescent lentiviral reporter pseudotyped with the SARS-CoV-2 Spike glycoprotein, replacing the commonly used VSV-G envelope protein that binds cells via non-specific lipids. This approach limits viral entry to ACE2-expressing target cells and has been widely used for screening of antiviral compounds and studying host cell entry mechanisms [34].

**Figure 4:**
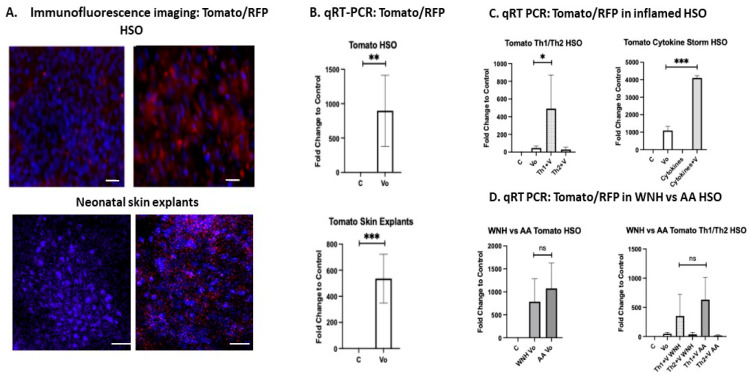
Neonatal skin explants and mature HSO were pretreated with vehicle control, cytokine storm-related cytokines (TNF-α + IL-6 + IL-1β + IFN-γ), Th1, or Th2 cytokines for 3 days. Tomato-expressing lentivirus pseudotyped with the SARS-CoV-2 Spike protein (10 µL, 10^7^ TU /mL) was applied to the surface of the skin models (2 models/group; experiments were repeated three times). (**A**). Tomato/RFP fluorescence was detected 72 h post-transduction in HSO and neonatal skin explants pretreated with vehicle control (Control) or virus only (Vo). Cell nuclei were counterstained with DAPI. Images were acquired from the apical aspect of whole-mount skin explants or HSO epidermis separated from the collagen matrix; (**B**). Quantification of Spike-pseudotyped lentiviral reporter entry by qRT-PCR analysis of *Tomato/RFP* mRNA expression in control (C) and virus only (Vo) HSO samples and Skin Explants. (**C**). Quantification of entry by *Tomato/RFP* mRNA expression by qRT-PCR in “inflamed” HSO pretreated with cytokines before topical application of pseudotyped lentivirus. The groups: control (C), virus only (Vo), Th1 + virus (Th1 + V), Th2 + virus (Th2 + V), cytokine storm (cytokines) and cytokine storm + virus (cytokines + Vo). *RPL27* was used as a normalization control. Statistical analysis was performed using an unpaired two-tailed Welch’s *t*-test. Error bars represent mean ± SD. Statistically significant difference compared to control: *—*p* < 0.05; **—*p* < 0.02; ***—*p* < 0.01; ns—not significant. In panel (**A**) red color represents the expression of Tomato/RFP and blue color marks the nuclei of keratinocytes. In panels (**B**–**D**) combined results of three experiments are presented.

Topical application of the Spike-pseudotyped Tomato/RFP reporter to mature HSO cultures resulted in strong Tomato/RFP fluorescence within the epidermis 48–72 h post-transduction (Figure 4A). Similar entry patterns were observed in neonatal skin explants following topical transduction with the Spike-pseudotyped Tomato/RFP reporter (Figure 4A).

For quantitative analysis of transduction efficiency in control and inflamed skin models, we measured *Tomato/RFP* mRNA expression by qRT-PCR 72 h after topical viral application (Figure 4B). Reporter transduction rates were dramatically increased in HSO pretreated with the combination of TNF-α + IL-6 + IL-1β + IFN-γ that mimics the cytokine storm observed in severe COVID-19 patients. Th1 cytokine pretreatment also significantly increased reporter transduction efficiency, whereas Th2 cytokines did not (Figure 4C). This was consistent with the preferential induction of *ACE2* rather than *TMPRSS2* by Th1 cytokines in these models (Figure 3C). Interestingly, there was a correlation between cytokine-induced upregulation of *ACE2* (e.g., approximately 16-fold with cytokine storm–related combination, Figure 2B; and approximately 6-fold with Th1 cytokines, Figure 3C) and increased Spike-mediated entry in HSO (Figure 4C,D). No significant differences in reporter transduction rates were observed between AA and WNH inflamed HSO, possibly due to the limited number of individual skin models used in these experiments (Figure 4D).

### 2.4. Similarity Between Molecular Signatures of Inflamed HSO Transduced with SARS-CoV-2 Reporter and SARS-CoV-2 Infection in Lungs of COVID-19 Patients

**Figure 5:**
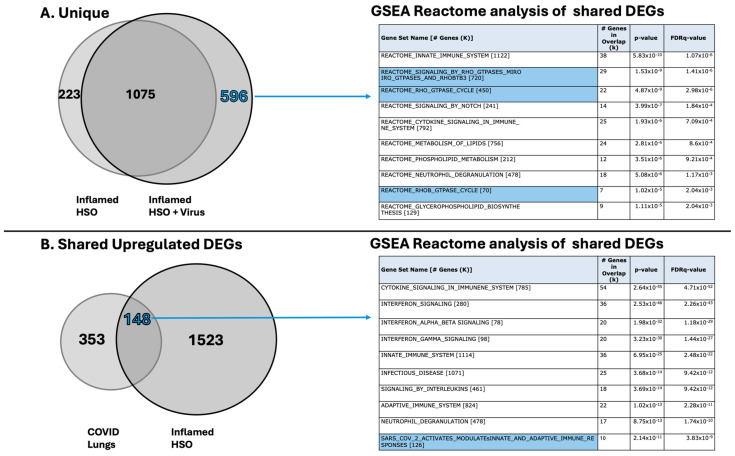
**Similarity Between Molecular Signatures of inflamed HSO transduced with SARS-CoV-2 Reporter and SARS-CoV-2 infection in Lungs of COVID-19 Patients**. RNA was extracted from the epidermal compartment of control HSO, HSO treated with the cytokine cocktail, and HSO transduced with Reporter after pretreatment with the cytokine cocktail (TNF-α + IL-6 + IL-1β + IFN-γ). RNA (two individual HSO per group) was subjected to bulk RNA sequencing. (**A**). Increased transcriptomic signature in inflamed HSO after transduction with SARS-CoV-2 Spike reporter. Differentially expressed genes (DEGs; FC > 2 and FC < 0.5, *p* < 0.05; FDR < 0.01) in HSO treated with cytokine cocktail compared to HSO pretreated with cytokine cocktail followed by Spike-pseudotyped lentiviral reporter entry; Reactome pathway enrichment analysis (GSEA) of unique 596 upregulated DEGs induced by Spike-pseudotyped lentiviral reporter entry with highlighted Rho GTPase signaling pathways. (**B**). Shared upregulated DEGs in HSO pretreated with cytokine cocktail followed by Spike-pseudotyped lentiviral reporter entry and DEGs identified in lungs of deceased COVID-19 patients (top 500 upregulated DEGs, GSE150316). Reactome pathway enrichment analysis (GSEA) of overlapping 148 DEGs, with highlighted pathway related to SARS-CoV-2 infection.

To further assess the clinical relevance of our skin model for SARS-CoV-2 infection, we compared gene expression signatures from lungs of deceased COVID-19 patients (GSE150316 presented at https://maayanlab.cloud/Enrichr/ site generated by Dr. Ma’ayan’s Laboratory at Mount Sinai Center for Bioinformatics (NYC), with transcriptomic profile of HSO treated with the cytokine storm–related cytokine combination typical for patients with severe COVID-19 and subsequently transduced with the SARS-CoV-2 Spike reporter [29,30] (Figure 5). The URL https://maayanlab.cloud/covid19/genesets/891 was accessed on 4 April 2025.

Because fluorescent reporter (such as GFP or RFP) expression has been shown to have minimal effects on key cellular metabolic pathways and does not broadly alter host transcriptional programs [35,36], we assumed that transcriptomic changes observed in transduced skin organoids primarily reflect downstream signaling induced by Spike protein binding to keratinocytes ACE2 in an inflamed HSO, rather than cell response to reporter DNA or expression of RFP.

RNA isolated from control HSO, cytokine-treated HSO, and cytokine-inflamed HSO transduced with the SARS-CoV-2 reporter lentivirus was subjected to bulk RNA sequencing (GSE304119), and differentially expressed genes (DEGs, FC > 2 and FC < 0.5; *p* < 0.01, FDR < 0.05) were identified. As expected, the combination of inflammatory cytokines induced substantial transcriptomic changes in HSO (2473 up- and downregulated DEGs). Notably, transduction with the reporter lentivirus further broadened the cytokine-induced response, increasing the total number of DEGs to 3383 (Figure 5A). Gene set enrichment analysis (GSEA) of 596 uniquely upregulated DEGs induced by the reporter virus in inflamed HSO revealed strong enrichment of pathways related to inflammation, including innate immune responses, cytokine signaling, and neutrophil degranulation, as well as multiple pathways associated with the Rho (Ras homolog family) GTPase cycle (Figure 5A, GSEA table). These are important and interesting findings as Rho GTPases, the key regulators of actin cytoskeleton dynamics and cellular architecture [37], play critical roles in viral infection including SARS-CoV-2 infection by facilitating viral entry, promoting replication, and regulating vesicular transport processes required for viral assembly and release [38,39]. Consistent with this, recent phosphoproteomic analyses of host cells infected with different SARS-CoV-2 variants has demonstrated the increased expression and activation of Rho GTPases as one of the major changes after infection [39].

Next, we compared the transcriptomes of lungs from deceased COVID-19 patients with the transcriptomic changes induced in cytokine-inflamed HSO following reporter lentivirus transduction. DEGs lists in lung tissue of patients with COVID-19 were obtained from publicly available datasets curated within Enrichr, a comprehensive gene set enrichment analysis web server https://maayanlab.cloud/Enrichr/), developed by Dr. Ma’ayan laboratory at Mount Sinai Center for Bioinformatics (Mount Sinai Center, New York) [40]. The URL https://maayanlab.cloud/covid19/genesets/891 was accessed on 4 April 2025. 

Comparison of top 500 upregulated DEGs from COVID-19-infected lung tissues available on Enrichr webserver, with all 1671 upregulated DEGs from cytokine-treated, Spike-pseudotyped lentivirus-transduced skin organoids revealed a substantial shared transcriptional signature comprising 148 overlapping genes (Figure 5B). GSEA of these common DEGs identified significant enrichment of pathways related to inflammation, innate immune responses, and adaptive immune activation. Importantly, the analysis also revealed the gene set associated with SARS-CoV-2–induced activation of immune pathways indicating some similarity between pulmonary COVID-19 signature and the signaling driven by Spike-pseudotyped lentiviral reporter in inflamed HSO models. Given that the lentiviral reporter system models viral entry does not recapitulate full viral replication, these transcriptional changes likely reflect host responses to Spike–ACE2 interaction and downstream signaling. Overall, the transcriptome changes in HSO after transduction with the SARS-CoV-2 reporter lentivirus further confirms the relevance of our models and supports the importance of studying skin responses to SARS-CoV-2 infection.

## 3. Discussion

The role of skin as a potential entry site for SARS-CoV-2 remains incompletely understood. In this study, we used in vitro skin models to assess whether a lentiviral reporter pseudotyped with SARS-CoV-2 Spike protein can attach to and enter keratinocytes within human epidermis. Both HSO and neonatal skin explants exhibit stratified epidermis with morphological features consistent with barrier formation, including a compact stratum corneum and dense stratum granulosum (Figure 1A; refs. [24,32]), as well as expression of tight junction markers such as ZO-1, occludin, and claudin-4, as previously reported [41,42]. However, neonatal skin and HSO possess a less mature epidermal barrier than intact adult skin in vivo [43,44]. This likely facilitated the observed entry of Spike-pseudotyped lentivirus in our experiments. Therefore, these results do not suggest that intact adult skin represents a primary physiological route of SARS-CoV-2 entry.

We observed robust expression of key viral entry factors—ACE2 and TMPRSS2 in 3D HSO and skin explants, consistent with studies demonstrating that epithelial differentiation strongly affects ACE2 expression [9,11]. This was important given conflicting reports in keratinocytes growing in monolayer: for example, Hertereau et al. [31] reported minimal ACE2 and no TMPRSS2 expression in 2D keratinocyte cultures. These findings highlight the importance of tissue context when evaluating susceptibility to viral entry.

Our data further indicate that inflammatory signaling reshapes the molecular landscape relevant to viral attachment and entry. Cytokines associated with severe COVID-19 and acute/chronic skin inflammation (TNF-α, IL-1β, IL-6, IFN-γ) significantly upregulated ACE2 and TMPRSS2, with synergistic effects in combination. Th1 cytokines (TNF-α + IL-17) preferentially induced ACE2, whereas Th2 cytokines (IL-4 + IL-13) increased TMPRSS2. These observations are consistent with prior reports of interferon-dependent ACE2 induction [9,31,45,46] and cytokine-mediated regulation of TMPRSS2 [46,47]. Functionally, these changes were associated with increased Spike-mediated virus entry into keratinocytes, particularly under Th1-skewed and cytokine storm–like conditions, while Th2 cytokines had minimal effects, consistent with their limited or even negative impact on ACE2 expression (Figure 3 and ref. [48]).

Transcriptomic analysis further revealed the biological relevance of the HSO model. Spike-mediated entry in inflamed HSO induced gene expression changes that partially overlap with signatures reported in lung tissue from COVID-19 patients, particularly in pathways related to innate immunity and inflammation. Although incomplete and exploratory because of the limited sample number, the observed overlap suggests that Spike–ACE2 engagement in keratinocytes may initiate downstream responses resembling aspects of host signaling studied in vivo and supports shared pathway-level enrichment. However, these findings obviously do not establish equivalence between reporter entry in skin models and pulmonary SARS-CoV-2 infection.

Several important limitations should be considered. First, the Spike-pseudotyped lentiviral reporter models viral entry but not the full viral life cycle. Second, both neonatal skin and HSO exhibit a less mature and functionally weaker barrier than adult human skin [43,44], which likely facilitated lentiviral reporter entry in our experiments. We used these in vitro models to show that keratinocytes possess all necessary machinery required for entry of SARS-CoV-2. Therefore, our results do not support the conclusion that intact adult skin represents a significant route of SARS-CoV-2 infection under normal physiologic conditions. Rather, the data suggests that under specific settings, such as impaired barrier integrity and/or inflammatory signaling, viral entry into epidermal cells may become more permissive. Accordingly, inflammatory skin diseases, barrier disruption, or anatomical sites with thinner or more permeable epidermis (for example, the eyelids) may represent conditions under which skin could transiently serve as a potential interface for SARS-CoV-2 host interactions. In addition, SARS-CoV-2 entry is not solely dependent on ACE2 and TMPRSS2; alternative receptors, cofactors, and proteases which are expressed in keratinocytes, may contribute to viral attachment and entry under certain conditions [49,50], although these factors (except CTSL) were not significantly induced by inflammatory cytokines in our study. We did not directly assess ACE2 and TMPRSS2 protein expression in adult human skin in this study, as adult skin biopsies or explants were not utilized. However, published studies reported detectable expression of ACE2 in the epidermis of adult human skin and its upregulation under inflammatory or mechanical stress conditions [51,52].

We also explored potential differences between African American and White non-Hispanic skin models. The relatively small sample size and inter-individual variability in ACE2 and TMPRSS2 expressions limited our ability to detect differences in susceptibility. Although a trend toward stronger Th1-induced ACE2 expression was observed in AA-derived HSOs, this did not translate into significant differences in viral entry. These results should be interpreted cautiously and are consistent with evidence that disparities in COVID-19 outcomes are largely driven by social and structural factors rather than intrinsic biological differences [25,26,27].

In summary, our study shows that inflammatory cues enhance expression of SARS-CoV-2 entry factors and increase susceptibility to Spike-mediated entry of the reporter virus in human skin models. These findings identify inflammation and barrier status as key modifiers of increased epidermal susceptibility to SARS-CoV-2. Rather than establishing skin as a primary entry route, our results suggest that, under conditions of barrier impairment or inflammation, the skin may represent a transient, limited and context-dependent interface for SARS-CoV-2 host interactions.

## 4. Materials and Methods

### 4.1. Human Skin Explants

Neonatal foreskin samples provided by the SBDRC STEM Core were obtained from routine circumcisions performed at Prentice Women and Children’s Hospital in Chicago. Grouping into AA and WNH categories was based on mothers’ self-reported ethnicity. Published data indicate that self-reported AA ancestry correlates with genotyping at approximately 85–90% [53].

### 4.2. 3D Human Skin Organoids (HSO)

HSO provided by the SBDRC STEM Core were prepared using normal human epidermal keratinocytes (NHEKs) isolated from AA and WNH neonatal foreskins, as previously described [28]. Grouping was based on mothers’ self-reported ethnicity. HSO were cultured at the air–liquid interface for 10–12 days prior to treatment to promote keratinocyte differentiation and skin barrier establishment. In each experiment we used HSO generated from several independent primary keratinocyte lines.

### 4.3. Treatment with Cytokines

Mature HSO with an established epidermis were treated for 7 days with individual cytokines: IL-1β, IL-6, IFN-γ, TNF-α (10 ng/mL each) or with their combination. In additional experiments, HSO were treated for 7 days with Th1 (IL-17 + TNF-α, 10 ng/mL each) or Th2 (IL-4 + IL-13, 30 ng/mL each) cytokine cocktails. Fresh cytokines were added every second day during media changes. The cytokines were purchased from R&D Systems (Minneapolis, MN, USA).

### 4.4. Transduction with SARS-CoV-2 Spike Protein-Pseudotyped Reporter Lentivirus

Standard lentiviral vectors are typically pseudotyped with vesicular stomatitis virus glycoprotein (VSV-G), which mediates viral entry through binding to widely expressed phospholipids rather than specific host receptors. To achieve ACE2-dependent cell entry, we generated a Tomato-expressing reporter lentivirus pseudotyped with the SARS-CoV-2 Spike protein by replacing VSV-G with the Spike protein from the Wuhan-Hu-1 strain as described [34].

HSO and skin explants were pretreated for four days with vehicle control (0.1% BSA in PBS) or with cytokine cocktails added to the culture medium: TNF-α + IL-1β + IL-6 + IFN-γ, Th1, or Th2 cytokines. Following pretreatment, HSO or skin explants were transduced by topical application of the SARS-CoV-2 Spike-pseudotyped Tomato (Red fluorescent protein, RFP) reporter lentivirus. Samples were harvested three days post-transduction, an optimal time point for detection of Tomato fluorescence. HSO were either immediately used for imaging and quantitative fluorescence analysis after cell nuclei were counterstained with DAPI for 10 minutes (Thermo Fisher Scientific, Waltham, MA, USA) or flash-frozen for downstream biochemical analyses of *Tomato* (*RFP*) expression at the mRNA level. 

### 4.5. Protein Extraction and Western Blotting

Neonatal foreskin samples were homogenized using a ceramic bead homogenizer (PRO250) followed by centrifugation for 1 min at 8000× *g*. HSO epidermis was homogenized by syringing. Proteins were extracted using Urea Sample Buffer (USB), consisting of consisting of 80% deionized Urea, 1% SDS, 10% glycerol, 0.06M Tris HCl (pH 6.8) and 0.001% Pyronin Y. Protein concentration was determined by Amido Black Assay on a VICTOR X plate reader (PerkinElmer, Shelton, CT, USA).

Proteins were resolved by SDS-PAGE and transferred to nitrocellulose membranes. The membranes were blocked by using 5% non-fat milk (Bio-Rad, Hercules, CA, USA) in Tris-Buffered Saline with Tween-20 (TBST) and incubated overnight at 4 °C with primary antibodies followed by secondary antibodies incubation for 1 h at room temperature. All washes between antibodies were done in TBST for 15 min three times. Membranes were imaged using AZURE ECL reagents and the AZURE Chemidoc digital imager (AZURE Biosystems, Dublin, CA, USA). GAPDH antibodies were used to normalize for equal sample loading. Membranes were re-probed with GAPDH antibodies after incubating in Stripping Buffer (Bio-Rad, Hercules, CA, USA) for 20 min, washing twice with TBST and blocking with 5% non-fat milk for 60 min. The differences in the protein expression between control and treated HSO were assessed by unpaired two-tailed Welch’s *t*-test.

We used following antibodies for Western blot analyses: ACE2 (Cell Signaling #4355S, Cell Signaling, Danvers, MA, USA), TMPRSS2 (#sc515727, Santa Cruz, Dallas, TX, USA), GAPDH (Cell Signaling #2118S, Cell Signaling, Danvers, MA, USA).

### 4.6. RNA Extraction and qRT-PCR

Skin samples were homogenized by PRO250 homogenizer (Pro Scientific, Oxford, CT, USA) on ice followed by syringing in QIAzol (Qiagen) buffer. HSO epidermis was homogenized by syringing in QIAzol buffer. RNA was extracted following the miRNeasy kit (Qiagen, Germantown, MD, USA) protocol. RNA concentrations were analyzed using a Nanodrop 2000 (Thermo-Fisher, Waltham, MA, USA) or RNA Qubit analysis (NUSeq Core, Northwestern University). cDNA synthesis was performed using random hexamers and M-MLV Reverse Transcriptase (Invitrogen, Carlsbad, CA, USA) according to the manufacturer’s protocol. The qRT-PCR for target genes was performed on a LightCycler 96 system (Roche Life Science, Indianapolis, IN, USA) with SYBR Green I PCR Master Mix (Roche Life Science, Indianapolis, IN, USA). House-keeping gene *RPL27* was used for normalization, relative expression was calculated by the standard 2^−ΔΔCt^ approach [54]. Primers were designed using NCBI BLAST (V 2.16) (http://www.ncbi.nlm.nih.gov/tools/primer-blast was accessed on 5 June 2024). The differences in the expression between control and treated AA and WNH samples were assessed by using unpaired two-tailed Welch’s *t*-test.

### 4.7. RNA-Sequencing and Analysis of Data

RNA was isolated from the epidermal compartment of control HSO, HSO treated with the cytokine cocktail, and HSO transduced with Reporter after pretreatment with the cytokine cocktail (TNF-α + IL-6 + IL-1β + IFN-γ); two HSO/group. RNA-seq was performed by LC Sciences (Houston, TX, USA) on the Illumina Novaseq™ 6000 (Illumina, San Diego, CA, USA) platform (paired-end reads, 150 bp). Per-base quality was assessed with FastQC software (V0.12.0) (http://www.bioinformatics.babraham.ac.uk/projects/fastqc/ was accessed on 10 April 2025). Alignment of fastq files to the human genome (ftp://ftp.ensembl.org/pub/release-96/fasta/homo_sapiens/dna/ accessed on 6 April 2026) was done with HISAT2. The assembly of mapped reads of each sample and estimation of the expression levels was performed using StringTie (version 2.2.1). The results of RNA-seq were deposited at NCBI (GSE304119). DESeq2 was used to perform differential expression analysis. DEGs were selected at *p*-value ≤ 0.01 (FDR < 0.05) and FC > 2 or FC < 0.5. GSEA analysis was performed online using Molecular Signatures Database (MSigDB) v2026.1. Hs updated in January 2026, a joint project of UC San Diego and Broad Institute [55].

The list of differentially expressed genes (DEGs) from lungs of patients with COVID-19 was obtained from Enrichr, a gene set analysis platform developed by Dr. Ma’ayan’s Laboratory at Mount Sinai Center for Bioinformatics, New York. The list is called “Top 500 up genes from SARS-CoV-2 infection in human lung from GSE150316” and could be accessed at https://maayanlab.cloud/covid19/genesets/891. The original GSE150316 deposition is “Spectrum of Viral Load and Host Response Seen in Autopsies of SARS-CoV-2 Infected Lungs” by Desai et al. (Massachusetts General Hospital, Boston, MA, USA). These COVID-19 lung DEGs, provided as curated gene lists within Enrichr, were compared with RNA-seq data generated from HSO treated with cytokine storm-associated cytokines and transduced with lentivirus pseudotyped with the SARS-CoV-2 spike protein. The URL https://maayanlab.cloud/covid19/genesets/891 was accessed on 4 April 2025.

### 4.8. Multiphoton Microscopy Imaging of Tomato-SARS-CoV-2 Spike-Pseudotyped Reporter Transduction in HSO and Explant Samples

Imaging was conducted using a Nikon A1R-MP microscope at the Northwestern Nikon Imaging Center. HSO samples were collected post-transduction, mounted on charged slides, rinsed with PBST buffer, treated with 4′,6-diamidino-2-phenylindol (DAPI) for 10 min for nuclear staining and covered with glass coverslips in Gelvatol to prevent fluorescent signals fading. Neonatal skin explants were collected post-transduction, permeabilized using 0.1% Triton X-100, stained for 20 min by DAPI and mounted to glass slides in Gelvatol. Image analysis was conducted using the associated Nikon NIS-Elements Software and image processing software Image J (FIJI2)(V 1.54).

### 4.9. Statistical Analysis

In all experiments, statistical analysis of protein and mRNA expression was performed using an unpaired two-tailed Welch’s *t*-test. For cytokine-induced ACE2 and TMPRSS2 expression (Figure 2 and Figure 3), 6–8 HSO per group were analyzed. For transduction experiments with Spike-pseudotyped lentiviral reporter (Figure 4 and Figure 5), two HSO or skin explants per group were used for each experiment. The experiments with analysis of Tomato expression were repeated three times with independent cell lines.

## Data Availability

All raw and preprocessed RNA-Seq data used in this study were deposited in the GEO database with the GEO accession number GSE304119. All other data generated or analyzed during this study are included in this article.

## Supplementary Materials

The following supporting information can be downloaded at: https://www.mdpi.com/article/10.3390/ijms27125382/s1.

## Abbreviations

The following abbreviations are used in this manuscript:

AA	African American
ACE2	angiotensin converting enzyme 2
AD	atopic dermatitis
BSA	bovine serum albumin
COVID-19	coronavirus disease 2019
CTSL	Cathepsin L
DAPI	4′,6-diamidino-2-phenylindole
IFNγ	interferon gamma
IL-1β	Interleukin-1β
HSO	human skin organoid
NHEK	normal human epidermal keratinocytes
PBST	Phosphate-buffered Saline with Tween-20
RFP	red fluorescent protein
SARS-CoV-2	severe acute respiratory syndrome coronavirus 2
SDS-PAGE	Sodium Dodecyl Sulfate–Polyacrylamide Gel Electrophoresis
TBST	Tris-Buffered Saline with Tween-20
TMPRSS2	Transmembrane serine protease 2
TU	Transduction units
TNFa	Tumor Necrosis Factor Alpha
USB	Urea Sample Buffer
VSV-G	vesicular stomatitis virus glycoprotein
WNH	White non-Hispanic
