# Supplementary material for: Skin as a Potential Entry Point for SARS-CoV-2 Virus
Source: Int J Mol Sci. 2026 Jun 15;27(12):5382. doi: 10.3390/ijms27125382 (PMC13299263; doi:10.3390/ijms27125382)
Supplement: Supplementary file 1 [file ijms-27-05382-s001.zip › ijms-4274494-supplementary.pdf]

Table S1

Effect of inflammatory cytokines on expression of Spike-protein receptors and proteases implicated in SARS-CoV-2 virus host cells entry

Gene_ID	Gene_Name	Description	FC	Pval
ENSG00000130234	ACE2	angiotensin I converting enzyme 2	6.05	0.01
ENSG00000184012	TMPRSS2	transmembrane serine protease 2	18.25	0.01
ENSG00000135047	CTSL	cathepsin L	2.34	0.02
ENSG00000183762	KREMEN1	kringle containing transmembrane protein 1	0.27	0.03
ENSG00000141505	ASGR1	asialoglycoprotein receptor 1	0.17	0.02
ENSG00000099250	NRP1	neuropilin 1	2.05	0.45
ENSG00000140564	Furin	furin, paired basic amino acid cleaving enzyme	1.48	0.48
ENSG00000167601	AXL	AXL receptor tyrosine kinase	1.88	0.52

Table S1 legend:

Mature HSO (4/group made from the individual keratinocyte cell lines) were treated with combination of severe COVID-19 cytokine storm-related cytokine combination TNF-α+ IL-6+IL-1β+ IFN-γ (10 ng/mL each) for 7 days. RNA was extracted and used for bulk RNA-sequencing as described in M&M. FC – change in expression treated VS control; Pval – statistical significance of changes.
